# A Dilemma for Coherence Calculation: Should Preprocessing Filters Be Applied?

**DOI:** 10.3389/fnins.2022.838627

**Published:** 2022-02-10

**Authors:** Maoqi Chen, Zhiyuan Lu, Ping Zhou

**Affiliations:** Faculty of Rehabilitation Engineering, University of Health and Rehabilitation Sciences, Qingdao, China

**Keywords:** coherence, EEG, EMG, filter, power spectrum estimation

## Introduction

Coherence is often used to measure the connectivity between a pair of signals. It indicates how closely they are statistically related, or how much influence the two activities have on one another. For example, calculation of the coherence between electroencephalogram (EEG) and electromyogram (EMG) signals can be used to examine functional connections between human brain and muscles (Siemionow et al., 2010; Coffey et al., 2021). Calculation of the coherence between EMG signals from two different muscles is often used to evaluate the common synaptic input to their motor neuron pools (Keenan et al., 2012; Aguiar et al., 2018).

In practice, the signals are often preprocessed to suppress contaminating noise (such as baseline wandering and power interference) before calculating their coherence. For example, in Chen et al. (2018), surface EMG signals were collected using a Bagnoli EMG system (Delsys Inc., Boston, USA) with a built-in bandpass filter at 20–450 Hz. In Grosse and Brown (2003), surface EMG signals were band-pass filtered between 53 and 1,000 Hz. In Aguiar et al. (2018), surface EMG signals were band-pass filtered between 30 Hz and 1 kHz. In contrast, the commonly studied frequency bands in coherence analysis include 1–4 Hz (delta band), 4–8 Hz (theta band), 8–12 Hz (alpha band), 13–30 Hz (beta band), and over 30 Hz (gamma band) oscillations (Mima and Hallett, 1999; Liu et al., 2019; Hallett et al., 2021), which are often within the stop band of preprocessing filters. Under such circumstances, one may intuitively assume that the coherence in these bands cannot be revealed. However, this is not necessarily the experimental observations. For example, a significant coherence was found in alpha band even the surface EMG signals went through a system built-in high pass filter with cutoff frequency at 20 Hz (Chen et al., 2018). In van Asseldonk et al. (2014), a significant coherence was observed at low frequencies (below 10 Hz) when the raw EMG signals preprocessed by a 10-Hz high pass filter were used for coherence calculation.

Given the above, it remains ambiguous whether or how coherence calculation might be affected by a preprocessing filter, particularly for the stopband of the filter. We, therefore, explore this question from both theoretical and practical points of view, toward better understanding coherence calculation.

## Theoretical Analysis

The coherence function between two real-number ergodic random signals *x*(*t*) and *y*(*t*) is defined as:


(1) $$\begin{array}{l} C_{xy}\left(\omega \right)=\frac{{\left|G_{xy}\left(\omega \right)\right|}^{2}}{G_{xx}\left(\omega \right)G_{yy}\left(\omega \right)} \end{array}$$

where *G*_*xy*_(ω) is the cross-spectral density between *x*(*t*) and *y*(*t*), *G*_*xx*_(ω) and *G*_*yy*_(ω) represent the auto-spectral density of *x*(*t*) and *y*(*t*), respectively. *G*_*xy*_(ω) is the Fourier transform of the cross-correlation function:


(2) $$\begin{array}{l} R_{xy}\left(\tau \right)=E\left\{x\left(t\right)y\left(t+\tau \right)\right\} \end{array}$$

Expanding the auto-spectral density and the cross-spectral density by using the relationship between correlation function and convolution, *C*_*xy*_(ω) can be expressed as:


(3) $$\begin{array}{l} C_{xy}\left(\omega \right)=\frac{{\left|E\left\{X\left(-\omega \right)Y\left(\omega \right)\right\}\right|}^{2}}{E\left\{X\left(-\omega \right)X\left(\omega \right)\right\}\cdot E\left\{Y\left(-\omega \right)Y\left(\omega \right)\right\}}=\\ \;\frac{{\left|E\left\{X\left(-\omega \right)Y\left(\omega \right)\right\}\right|}^{2}}{E\left\{{\left|X\left(\omega \right)\right|}^{2}\right\}\cdot E\left\{{\left|Y\left(\omega \right)\right|}^{2}\right\}} \end{array}$$

where *X*(ω) and *Y*(ω) represent the Fourier transform of *x*(*t*) and *y*(*t*), respectively.

Now suppose *x*(*t*) and *y*(*t*) pass through two linear time-invariant (LTI) systems with impulse response *h*_1_(*t*) and *h*_2_(*t*) (not necessarily the same), respectively, and obtain the filtered signals *x*_*f*_(*t*) and *y*_*f*_(*t*), i.e., *x*_*f*_(*t*) = *h*_1_(*t*) * *x*(*t*) and *y*_*f*_(*t*) = *h*_2_(*t*) * *y*(*t*), where * denotes convolution. Then we have the following equations in the Fourier domain: *X*_*f*_(ω) = *H*_1_(ω)*X*(ω) and *Y*_*f*_(ω) = *H*_2_(ω)*Y*(ω), where *H*_1_(ω) and *H*_2_(ω) are the Fourier transform of *h*_1_(*t*) and *h*_2_(*t*), respectively. For convenience, we assume that *H*_1_(ω) and *H*_2_(ω) are not 0 at any frequency ω.

The following mathematical derivation justifies that in theory, the coherence between *x*_*f*_(*t*) and *y*_*f*_(*t*) is the same as the coherence between *x*(*t*) and *y*(*t*).


(4) $$\begin{array}{l} C_{x_{f}y_{f}}\left(\omega \right)=\frac{{\left|E\left\{X_{f}\left(-\omega \right)Y_{f}\left(\omega \right)\right\}\right|}^{2}}{E\left\{{\left|X_{f}\left(\omega \right)\right|}^{2}\right\}\cdot E\left\{{\left|Y_{f}\left(\omega \right)\right|}^{2}\right\}}\\ \;=\frac{{\left|E\left\{H_{1}\left(-\omega \right)X\left(-\omega \right)H_{2}\left(\omega \right)Y\left(\omega \right)\right\}\right|}^{2}}{E\left\{{\left|H_{1}\left(\omega \right)X\left(\omega \right)\right|}^{2}\right\}\cdot E\left\{{\left|H_{2}\left(\omega \right)Y\left(\omega \right)\right|}^{2}\right\}}\\ \;=\frac{{\left|H_{1}\left(\omega \right)\right|}^{2}\cdot {\left|H_{2}\left(\omega \right)\right|}^{2}\cdot {\left|E\left\{X\left(-\omega \right)Y\left(\omega \right)\right\}\right|}^{2}}{{\left|H_{1}\left(\omega \right)\right|}^{2}\cdot E\left\{{\left|X\left(\omega \right)\right|}^{2}\right\}\cdot {\left|H_{2}\left(\omega \right)\right|}^{2}\cdot E\left\{{\left|Y\left(\omega \right)\right|}^{2}\right\}}\\ \;=\frac{{\left|E\left\{X\left(-\omega \right)Y\left(\omega \right)\right\}\right|}^{2}}{E\left\{{\left|X\left(\omega \right)\right|}^{2}\right\}E\left\{{\left|Y\left(\omega \right)\right|}^{2}\right\}}=C_{xy}\left(\omega \right) \end{array}$$

Note that the third step of Equation (4) holds because *H*_1_(ω) and *H*_2_(ω) are not random variables and therefore can be separated out from expectation. It is concluded from Equation (4) that a LTI system used for signal preprocessing will not affect calculation of coherence, as long as its frequency response function is non-zero at the frequency of interest.

## Practical Consideration

In practical application, it is difficult to accurately calculate coherence since in reality we only have finite samples. By assuming that the signal is ergodic, we usually use the Welch's method (Welch, 1967) to approximate coherence. Welch's method is a modified periodogram method for estimating auto-spectral density and cross-spectral density. Specifically, the signal is divided into *N* equal length segments (either overlapping or non-overlapping), and then *C*_*xy*_(ω) is estimated as:


(5) $$\begin{array}{l} C_{xy}\left(\omega \right)=\frac{{\left|\sum_{i=1}^{N}X_{i}\left(-\omega \right)Y_{i}\left(\omega \right)\right|}^{2}}{\left(\sum_{i=1}^{N}{\left|X_{i}\left(\omega \right)\right|}^{2}\right)\left(\sum_{i=1}^{N}{\left|Y_{i}\left(\omega \right)\right|}^{2}\right)} \end{array}$$

One should note that coherence is only meaningful for random signals, so segmentation is necessary. If the whole signal is used as one segment, the coherence will always equal to 1 regardless what the true value is.

Signal segmentation, however, inevitably imposes a windowing operation (such as a hamming window). Windowing in the time domain means convolution in the frequency domain, which will cause spectral leakage. The spectral leakage effect often induces spectral fluctuations in the area outside the spectral peaks. As a result, the spectral densities estimated by Welch's method usually have large variances. In fact, this adverse impact is unavoidable in the existing spectrum estimation methods based on the periodogram. In this case Equation (4) will not strictly hold. Therefore, the filtering operation, indeed, will have a certain impact in actual implementation of the coherence calculation. It is difficult to quantify this impact, as it largely depends on multiple factors such as specific window and filter characteristics.

## Dilemma

Based on the above analyses, it can be concluded that in theory a preprocessing LTI filter does not improve or influence coherence estimation. Therefore, it is not surprising to observe significant coherence in the stopband of the preprocessing filters (van Asseldonk et al., 2014; Chen et al., 2018). In practice, however, due to limitations of the existing power spectrum estimation methods, a LTI filter will unavoidably influence coherence estimation.

It remains a dilemma to determine whether the signals should be preprocessed by a LTI system before calculating the coherence in practical application. On one hand, if a preprocessing filter is not applied, the noise contaminating the signals will contribute to coherence calculation. On the other hand, if a preprocessing filter is applied, the filtering operation itself has a risk of distorting coherence calculation, especially for the stopband of the filter (likely due to those unpredictable factors such as quantization errors introduced during implementation of Welch's method). Unfortunately, it is difficult to quantitatively assess the detrimental effect imposed by the filtering operation or by the contaminating noise, in order to provide a preference for coherence calculation.

The main reason for this dilemma lies in the methodology limitation of the periodogram-based power spectrum estimation. Large variance is one of the disadvantages of Welch's method for estimating spectral density, which may result in unreliable coherence estimation (regardless of whether a LTI filter is applied). For example, there may be considerable false peaks that are difficult to distinguish. To overcome this limitation, a possible approach is to smooth the power spectrum curve using alternative estimation methods such as parametric models (e.g., AR model). However, the parametric method may produce unreliable results when the preset model cannot match the signal reasonably well.

## Summary

By theoretical analysis we prove that a LTI system will not affect calculation of coherence, as long as its frequency response function is non-zero at the frequency of interest. However, because of the methodology limitation of spectral estimation, a dilemma arises in practice regarding whether a preprocessing LTI filter should be applied before coherence calculation. How to overcome this dilemma needs further exploration and discussion, depending on specific circumstances of coherence calculation.

## Author Contributions

MC wrote the first draft of this opinion manuscript. All authors contributed to its conception and development, revised, and approved the final version.

## Funding

This work was supported in part by the Shandong Provincial Natural Science Foundation under grant nos. ZR2021QH053 and ZR2020KF012, and in part by the National Nature Science Foundation of China under grant no. 82102179.

## Conflict of Interest

The authors declare that the research was conducted in the absence of any commercial or financial relationships that could be construed as a potential conflict of interest.

## Publisher's Note

All claims expressed in this article are solely those of the authors and do not necessarily represent those of their affiliated organizations, or those of the publisher, the editors and the reviewers. Any product that may be evaluated in this article, or claim that may be made by its manufacturer, is not guaranteed or endorsed by the publisher.
